# High NUCB2 expression level represents an independent negative prognostic factor in Chinese cohorts of non-metastatic clear cell renal cell carcinoma patients

**DOI:** 10.18632/oncotarget.12961

**Published:** 2016-10-28

**Authors:** Hangcheng Fu, Yu Zhu, Yiwei Wang, Zheng Liu, Junyu Zhang, Zewei Wang, Huyang Xie, Bo Dai, Jiejie Xu, Dingwei Ye

**Affiliations:** ^1^ Department of Urology, Fudan University Shanghai Cancer Center, Shanghai, China; ^2^ Department of Oncology, Shanghai Medical College, Fudan University, Shanghai, China; ^3^ Department of Urology, Ninth People's Hospital, School of Medicine, Shanghai Jiaotong University, Shanghai, China; ^4^ Department of Biochemistry and Molecular Biology, School of Basic Medical Sciences, Fudan University, Shanghai, China

**Keywords:** renal cell carcinoma, NUCB2, prognostic factor, cancer-specific survival

## Abstract

**Background:**

This study aimed to investigate the prognostic significance of NUCB2 in clear cell renal cell carcinoma.

**Patients and Methods:**

The study retrospectively enrolled a training set (182 patients) and a validation set (434 patients) with non-metastasis (pT1-3N0M0) ccRCC from two institutional medical centers of China. NUCB2 protein expression was evaluated by immunohistochemical staining of NUCB2 antibody, and its association with clinicopathological characteristics and clinical outcomes were evaluated. The NUCB2 mRNA transcription level was evaluated through TCGA KIRC cohort (190 patients). Prognostic accuracies were evaluated by *C* index and Akaike information criterion.

**Results:**

In ccRCC tissues, NUCB2 protein expression level was positively correlated with Fuhrman grade (*P* = 0.002 and *P* < 0.001, respectively). Patients with high NUCB2 mRNA transcription level (*P* = 0.005) and protein expression level (*P* = 0.024 and *P* < 0.001, respectively) had shorter cancer-specific survival in Kaplan-Meier survival curve. Moreover, multivariate analysis identified NUCB2 expression level as an independent prognostic factor for cancer-specific survival. Subgroup analysis suggested that NUCB2 expression significantly stratified pT1 stage patients (*P* < 0.001) rather than higher pT stage patients. Therefore, a new NNF prognosis model was developed to predict the cancer-specific survival in patients with pT1N0M0 stage (*C*-index = 0.743).

**Conclusion:**

NUCB2 expression level is a powerful independent prognostic factor for CSS in patients with non-metastasis (pT1-3N0M0) ccRCC.

## INTRODUCTION

The incidence rates of renal cell carcinoma (RCC) have increased slightly within the last decades worldwidely, and a stage migration towards smaller and more organ-confined tumors has been observed [1, 2]. Despite of the early imaging detection and curative resection of the tumor, 20-30% of patients with non-metastatic ccRCC still experienced recurrence and metastasis [3]. And, worse still, the 5-year survival for patients is below 10% once malignancy metastasis. To predict the risk of disease development, currently, several clinicopathological prognostic models have been developed to predict clinical outcome for postoperative patients with clear cell renal cell carcinoma (ccRCC). Such models include the TNM staging system, the Leibovich score, the University of California Los Angeles Integrated Staging System (UISS) and the Mayo Clinic stage, size, grade, and necrosis (SSIGN) score [4–6]. Additionally, the 5-year CSS rate was 91% for T1N0M0 patients which comprised the largest group of non-metastatic ccRCC patients [7] while no specific prognostic algorithm was developed to distinct those patients with poor prognosis. Some recent studies combined these standard models with molecular biomarkers to make progress in better stratifying recurrence risk to aid clinical counselling, personalize follow-up, and target adjuvant treatment trails [8, 9]. Thus, to evaluate the prognostic value of genetic and proteome marker in patients with RCC and build prognostic algorithm in particular patients’ subgroup are still ongoing unremittingly.

NUCB2, as a precursor protein of nesfatin-1, was identified as a hypothalamus secreted protein originally and was found to be involved in feeding regulation in hypothalamic nuclei [10]. Subsequently, NUCB2 expression was found in peripheral tissues such as pancreatic islets, testis, stomach and adipose tissue [11, 12]. Recently, some studies proposed possible roles of NUCB2 in several human malignancies, which still remain controversial [13–19]. Nesfatin-1 has been reported to suppress cell proliferation of adrenocortical carcinoma cells and epithelial ovarian carcinoma [15, 16], while NUCB2 was shown to promote cell proliferation and invasion of breast carcinoma cells, and was associated with unfavorable clinical outcomes in prostate and breast cancer [13, 18]. As in the renal cell carcinoma, only one study which included 188 patients showed that NUCB2 expression was an indicator for worse prognosis in patients with ccRCC [20]. However, the study was based on a small cohort and the prognostic role of NUCB2 in different clinicopathological subgroup of patients was still unveiled. Therefore, the aim of this study was to further clarify the prognostic value of NUCB2 expression in ccRCC and identify the prognostic ability in distinctive subgroup of patients.

## PATIENTS AND METHODS

### Patients selection

The Research Medical Ethics Committee of Fudan University appoved this study. Two sets of patients with non-metastasis (pT1-3N0M0) clear cell renal cell carcinoma (ccRCC) from different institutional medical center were rolled independently in this study. 182 and 434 consecutive patients from Shanghai Cancer Center (Shanghai, China) and Zhongshan Hostipal (Shanghai, China) between 2008 and 2009 were included as training set and validation set, respectively. The primary inclusion criterion was (1) histopathologically proven non-metastasis ccRCC; (2) underwent radial- or partial- nephrectomy as therapeutic intervention without adjuvant treatment; (3) available tissue specimen of intratumoral mass (≥1cm^3^) with Formalin Fixed Paraffin Embedded (FFPE). Patients who died within the first month after the surgery or lost follow up; patients with positive surgical margin, unspecified tumor location, bilaterally kidney tumor or familial RCC were excluded. 12 patients in the training set and 17 patients in the validation set lost follow up. Clinical baseline data for each patient were gathered retrospectively. The tumor size was defined with the longest diameters of the samples. Histological subtype was re-stratified according to 2014 EAU guidelines. All specimens were reassessed independently by two uropathologists according to the 2010 AJCC TNM classification. SSIGN, UISS score and Leibovich category were applied to all patients according to original algorithm [4, 6]. We calculated the cancer-specific survival (CSS) from the day of surgery to the most recent follow-up or the day of tumor-related death. The median follow-up was 86 months and 70 months, respectively. The number of events were 28 and 48 in the training cohort and validation cohort, respectively.

A total of 190 patients with non-metastasis (T1-3N0M0) ccRCC from TCGA KIRC cohort were included in the study as well. The expression profile of NUCB2 and related genes were obtained from the TCGA RNAseq database. Clinicopathological characteristics, including age, gender, tumor size, tumor grade, T stage, ECOG, necrosis, and overall survival were also collected. The median of quantitative PCR of RNAseq (10.3) was used to differentiate low transcription level and high transcription level. The median follow-up was 38 months.

### Immunohistochemistry (IHC) and evaluation of immunostaining

Tissue microarray were constructed with duplicate 1.0 mm tissue cores from two distinctive areas. The mean score of the two spots was adopted. Immunohistochemistry study were then performed as described previously [21]. Rabbit anti-NUCB2 polyclonal antibody (Sigma, 1:100) was applied to identify intratumoral NUCB2 expression. The negative control was stained equally with the primary antibody omitted. Two independent uropathologists were blinded to the clinicopathological data and evaluated the staining intensity. A semi-quantitative H-score was computed for each sample by multiplying the staining intensities (0: negative, 1: weak staining, 2: moderate staining, 3: strong staining) and distribution areas (0-100%), which ranged from 0 to 300. The stroma or fat tissue was excluded when evaluating. The results of 5 (2.04%) cases experienced disagreement (H-score difference > 10) between the pathologists at first. Finally, the agreement scores were reached after full discussion.

The intraclass correlation was 0.995 (95% CI, 0.993 to 0.997; *P* < .0001) between the scores of two uropathologists. The intraclass correlation was 0.946 (95% CI, 0.937 to 0.953; *P* < .0001) between two cores. Median score (110) was used to dichotomize all specimens into low expression group and high expression group. The score of low expression group was 4-110 (*n* = 82) and 9-109 (*n* = 245) in the training set and validation set, respectively. The score of high expression group 110-245 (*n* = 100) and 110-285 (*n* = 189), respectively.

### Statistical analysis

Statistical analysis was conducted with Stata 12.0 (StataCorp, College Station, TX), MedCalc software (version 11.4.2.0; MedCalc, Mariakerke, Belgium) and R programming language version 3.2.2 (R Foundation for Statistical Computing, Vienna, Austria). CSS survival curves were calculated with Kaplan-Meier method and log-rank test. Numerical variables were analyzed by Student *t* test, and categorical variables were calculated by Chi-square test. Univariate and multivariate Cox proportional hazard models were performed to calculate hazard ratios (HR) and 95% confidence intervals (95% CI). Nomogram models were calculated with the “rms” package of R programming language. The prognostic accuracy of the prognostic models was assessed by Harell concordance index (C-index) and Akaike information criterion (AIC).

## RESULTS

### Immunohistochemical findings

**Figure 1:**
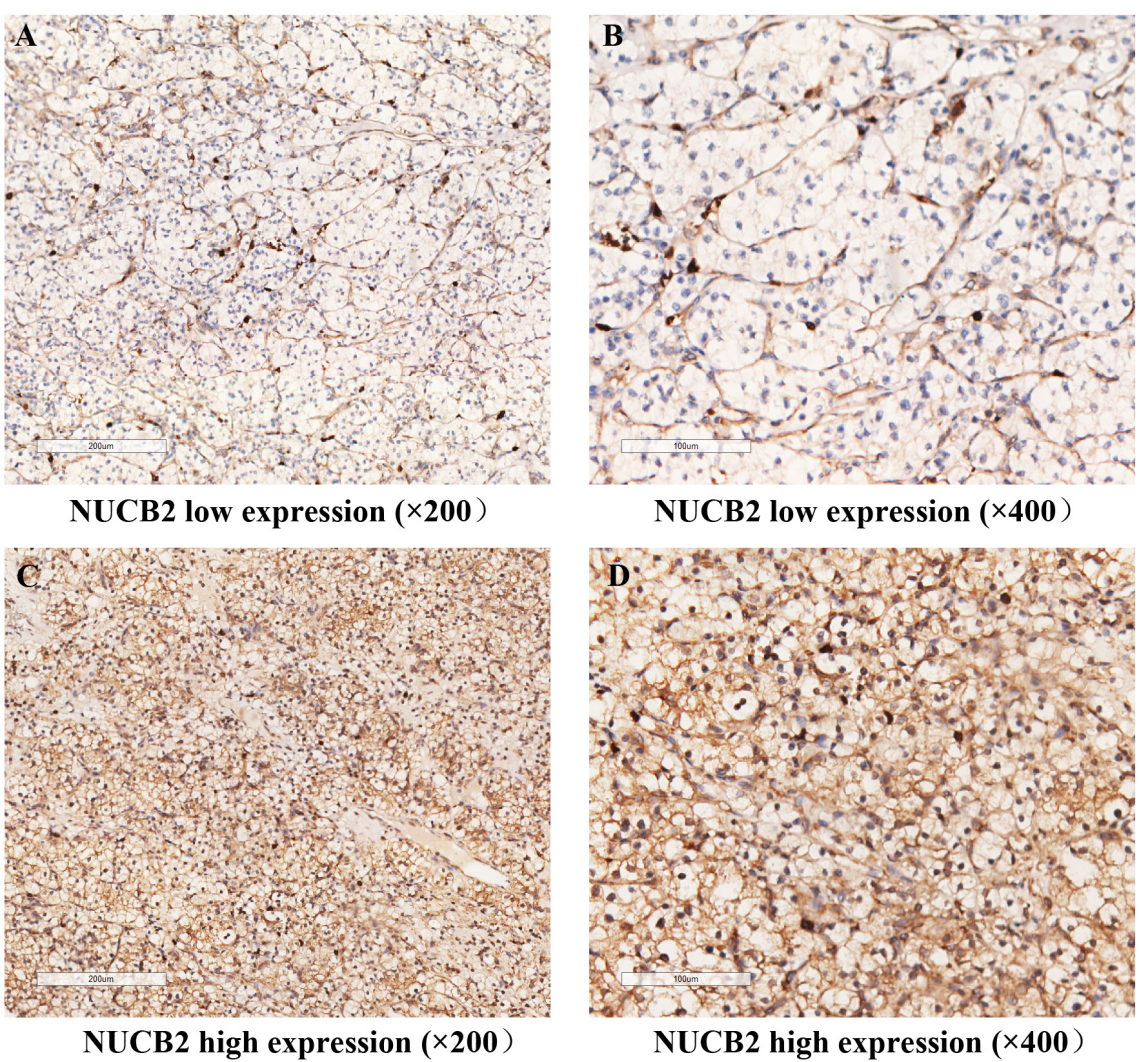
NUCB2 expression in clear-cell renal cell carcinoma (ccRCC) tissues Representative immunohistochemical images of low NUCB2 expression in ccRCC tissue at 200× optical magnification **A**. and 400× optical magnification **B**.. Representative immunohistochemical images of high NUCB2 expression in ccRCC tissue at 200× optical magnification **C**. and 400× optical magnification **D**..

To identify whether the expression of NUCB2 is related to the tumor progression of ccRCC, firstly NUCB2 expression was evaluated by immunohistochemical analysis in renal tumor tissue specimens from training set and validation set. As shown in Figure 1, NUCB2 was stained clear and distinguishable. The dichotomization between low expression group and high expression group was described before.

### Correlation of NUCB2 expression with clinicopathological factors of ccRCC patients

As summarized in Table 1, the patient's characteristics and NUCB2 expression from training set (*n* = 182), validation set (*n* = 434) and TCGA KIRC cohort (*n* = 190) were summarized in the study. The ECOG and tumor necrosis data of TCGA KIRC cohort were of incompleteness. NUCB2 expression was shown to be positively associated with higher Fuhrman grade both in the training cohort and validation cohort (*P =* 0.002 and *P =* 0.001, respectively). Furthermore, NUCB2 high expression was correlated with worse ECOG PS score (*P =* 0.004) and presence of necrosis (*P <* 0.001) in the validation set. In the TCGA KIRC cohort, higher mRNA transcription level was correlated with higher T stage (*P =* 0.017).

**Table 1:** Correlation between NUCB2 expression, transcription and patient characteristics

Characteristic	NUCB2 expression						NUCB2 mRNA transcription		
	Training cohort			Validation cohort			TCGA KIRC cohortc		
	Low (%)	High (%)	*P*^a^	Low (%)	High (%)	*P*^a^	Low	High	*P*^a^
All patients	82	100		245	189		95	95	
Age(years)^b^			0.258			0.852			0.177
Mean ± SD	54.7 ± 12.9	52.5 ± 12.8		55.3 ± 11.7	55.1 ± 12.7		61.3 ± 12.8	63.7 ± 11.4	
Median	53	53		55	55		62	64	
Range	29-78	20-83		30-86	22-85		34-86	38-90	
Tumor size(cm)^b^			0.284			0.352			0.105
Mean ± SD	4.6 ± 3.0	4.2 ± 2.1		4.1 ± 2.1	4.4 ± 2.7		0.9 ± 0.2	1.0 ± 0.3	
Median	4.0	3.8		4.0	4.0		0.9	1.0	
Range	1.0-20.0	1.5-12.5		0.2-14.0	0.5-15.0		0.3-1.8	0.4-2.0	
Gender			0.209			0.872			0.768
Female	23 (12.6)	38 (20.9)		71 (16.3)	57 (13.1)		41 (21.6)	38 (20.0)	
Male	59 (32.4)	62 (34.1)		174 (40.2)	132 (30.4)		54 (28.4)	57 (30.0)	
pT stage			0.516			0.107			**0.017**
T1	69 (37.9)	85 (46.7)		181 (41.7)	123 (28.3)		58 (30.5)	40 (21.1)	
T2	8 (4.5)	6 (3.3)		17 (3.9)	14 (3.2)		15 (7.9)	16 (8.4)	
T3	5 (2.7)	9 (4.9)		47 (10.8)	52 (12.1)		22 (11.6)	39 (20.5)	
Fuhrman grade			**0.002**			**<0.001**			0.098
1+2	71 (39.0)	65 (35.7)		186 (42.8)	95 (21.9)		54 (28.4)	46 (24.2)	
3	8 (4.4)	31 (17.0)		43 (9.9)	59 (13.6)		37 (19.5)	37 (19.5)	
4	3 (1.6)	4 (2.3)		16 (3.7)	35 (8.1)		4 (2.1)	12 (6.3)	
ECOG PS			0.241			**0.004**			0.979
0	69 (37.9)	76 (41.7)		217 (50.0)	147 (33.8)		9 (4.7)	18 (9.4)	
≥1	13 (7.2)	24 (13.2)		28 (6.5)	42 (9.7)		3 (1.6)	4 (2.1)	
Necrosis			0.422			**<0.001**			0.097
Absent	76 (41.7)	88 (48.3)		213 (49.2)	135 (31.1)		87 (45.8)	76 (40.0)	
Present	6 (3.3)	12 (6.7)		32 (7.3)	54 (12.4)		6 (3.2)	14 (7.4)	

ECOG PS = Eastern Cooperative Oncology Group performance status

^a^, *P* < 0.05 is considered statistically significant, *t*-test for continuous variables and χ2 test for categorical variables.

^b^, the results of continuous variables are presented as mean ± SD (standard deviation).

^c^, the data of ECOG PS and tumor necrosis were incomplete.

### Correlation between NUCB2 mRNA transcription, protein expression and clinical outcomes in patients with ccRCC

**Figure 2:**
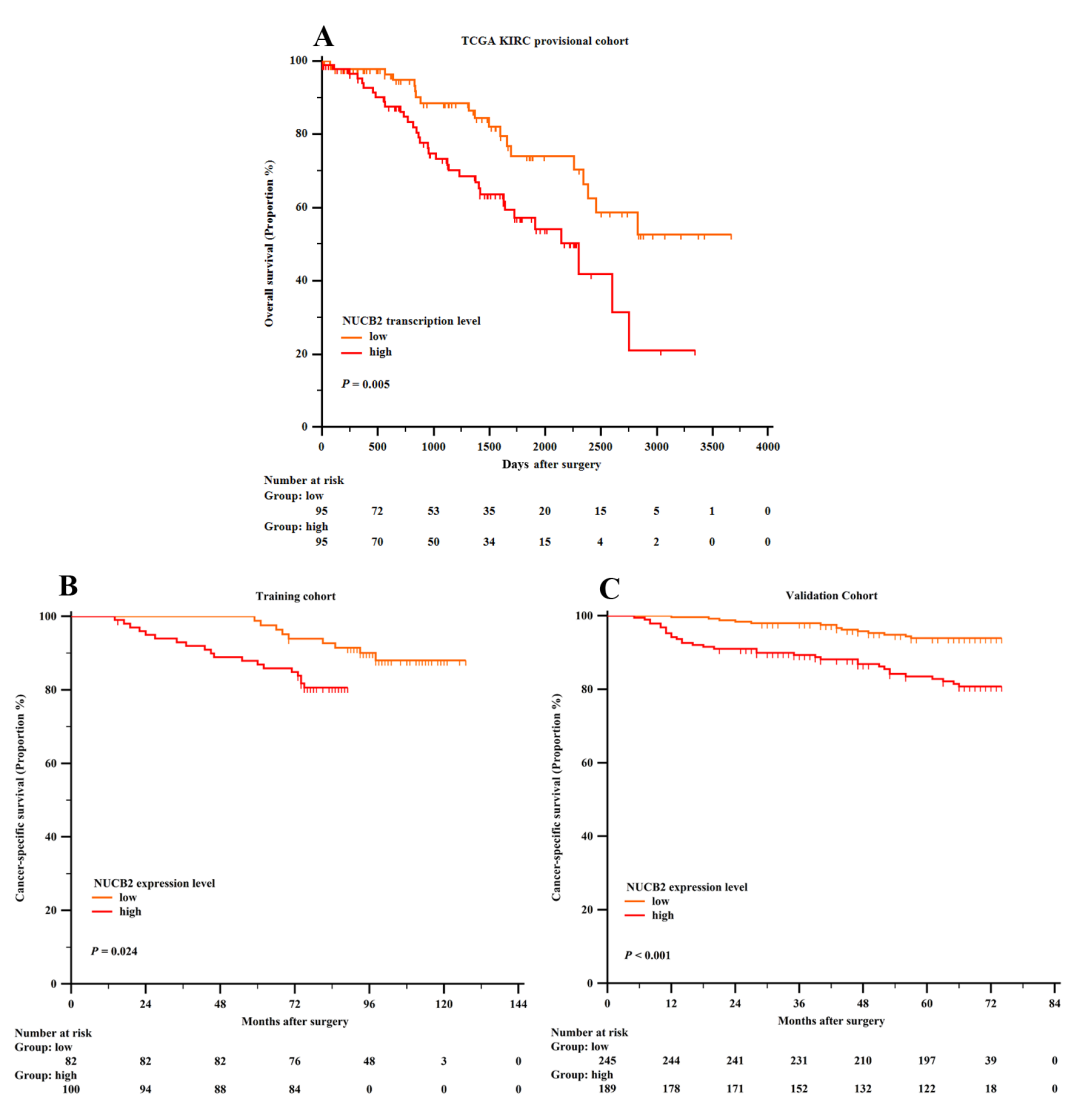
Kaplan-Meier analysis of cancer-specific survival of patients with clear-cell renal cell carcinoma (ccRCC) based on NUCB2 mRNA level and protein level **A**. Overall survival of NUCB2 mRNA transcription level in TCGA KIRC cohort. **B**. Cancer-specific survival of NUCB2 protein expression level in training cohort. **C**. Cancer-specific survival of NUCB2 protein expression level in validation cohort.

For further analysis, we studied the prognostic value between NUCB2 transcription level, expression level and patients’ clinical outcomes with Kaplan-Meier analysis. As shown in Figure 2, Cancer-specific survival (CSS) and overall survival (OS) was compared between subgroups with Log rank test. In the TCGA KIRC cohort, patients with high mRNA transcription showed worse clinical outcome (*P <* 0.001). Also, NUCB2 expression was associated with unfavorable prognosis both in the training and validation cohort (*P =* 0.024 and *P <* 0.001, respectively).

### Univariate and multivariate Cox proportional hazard analysis

To evaluate whether NUCB2 expression was an independent prognostic indicator, univariate and multivariate analysis for CSS were conducted in the training cohort and validation cohort. As indicated in Table 2, Univariate Cox regression analysis identified statistically significant clinicopathological factors correlated with CSS which were included to performed multivariate Cox regression analysis. Table 2 showed that tumor necrosis (*P =* 0.050) and NUCB2 expression (*P =* 0.044) were identified as independent prognostic factor in training cohort. Moreover, tumor size (*P <* 0.001), pT stage (*P =* 0.012), Fuhrman grade (*P <* 0.001), tumor necrosis (*P =* 0.001) and NUCB2 expression (*P =* 0.018) were demonstrated as independent prognostic indicator in validation cohort with CSS.

**Table 2:** Univariate and Multivariate Cox regression analysis of clinical characteristic and NUCB2 expression in Cancer-specific Survival

Characteristics	Training cohort				Validation cohort			
	Univariate		Multivariate		Univariate		Multivariate	
	HR (95% CI)	*P*^a^	HR (95% CI)	*P*^a^	HR (95% CI)	*P*^a^	HR (95% CI)	*P*^a^
Age(year)^b^	1.017 (0.988-1.047)	0.245			1.017 (0.993-1.041)	0.171		
Tumor size(cm) ^b^	1.148 (1.056-1.248)	**0.001**	1.159 (0.999-1.345)	0.052	1.514 (1.378-1.664)	**<0.001**	1.340 (1.179-1.523)	**<0.001**
gender (male vs female)	0.846 (0.392-1.827)	0.672			1.548 (0.772-3.102)	0.220		
pT stage		**<0.001**		0.053		**<0.001**		**0.012**
3 vs 1	3.827 (1.408-10.406)	**0.009**	1.206 (0.296-4.915)	0.794	3.029 (1.039-8.829)	**0.043**	0.965 (0.292-3.187)	0.954
4 vs 1	7.082 (2.706-17.259)	**<0.001**	3.318 (1.180-9.325)	**0.024**	4.189 (2.296-7.642)	**<0.001**	2.440 (1.116-5.338)	**0.026**
Fuhrman grade		**0.001**		0.269		**<0.001**		**<0.001**
3 vs 1+2	3.594 (1.620-7.973)	**0.002**	2.095 (0.841-5.219)	0.114	2.869 (1.291-6.376)	**0.010**	1.792 (0.770-4.170)	0.178
4 vs 1+2	5.279 (1.521-18.325)	**0.009**	1.387 (0.286-6.727)	0.686	12.717 (6.187-26.140)	**<0.001**	5.641 (2.549-12.483)	**<0.001**
ECOG PS (>=1 vs 0)	2.146 (0.975-4.728)	0.059			4.430 (2.429-8.081)	**<0.001**	1.521 (0.765-3.022)	0.233
Necrosis (present vs absent)	5.999 (2.712-13.273)	**<0.001**	2.901 (1.001-8.410)	**0.050**	5.086 (2.872-9.006)	**<0.001**	2.927 (1.528-5.607)	**0.001**
NUCB2	2.694 (1.128-6.431)	**0.026**	2.614 (1.033-6.616)	**0.044**	3.464 (1.859-6.454)	**<0.001**	2.335 (1.160-4.701)	**0.018**

CI=confidence interval; ECOG PS = Eastern Cooperative Oncology Group performance status;

^a^, *P*<0.05 is considered statistically significant.

^b^, calculated with continuous variable.

### Prognostic nomogram of patients and extension of prognostic models with NUCB2 for CSS

Moreover, prognostic nomograms were built *via* integrating the independent prognostic indicators from validation cohort in Table 2 for CSS. Tumor size, pT stage, Fuhrman grade, necrosis and NUCB2 expression level were included to build nomogram (Figure S1).

Also, the NUCB2 expression level were integrated into different prognostic models respectively to evaluate the prognostic power of NUCB2 in Table 3. The prognostic accuracy was investigated by concordance index (C index) and Akaike information criteria (AIC) analysis. The prognostic accuracy of each original prognostic model was improved after integrating NUCB2 expression level for CSS. Moreover, the nomogram built showed the highest accuracy (*C*-index 0.834, AIC 793.1).

**Table 3:** Prognostication comparison of built-up nomogram and other models in cancer-specific survival (CSS)

Model	C-index	AIC
NUCB2	0.637	915.2
T stage	0.667	897.6
T stage + NUCB2	0.745	879.1
UISS category	0.728	866.2
UISS category + NUCB2	0.773	854.5
SSIGN category	0.780	821.5
SSIGN category + NUCB2	0.805	808.4
Leibovich category	0.785	824.2
Leibovich category + NUCB2	0.810	809.9

Abbreviation: CSS, cancer-specific survival; RFS, relapse-free survival; C-index, concordance index; AIC, Akaike's information criterion; UISS, UCLA Integrated Staging System; 95% CI, 95% confidence interval.

### Subgroup analysis of NUCB2 expression upon different clinicopathological factors

**Figure 3:**
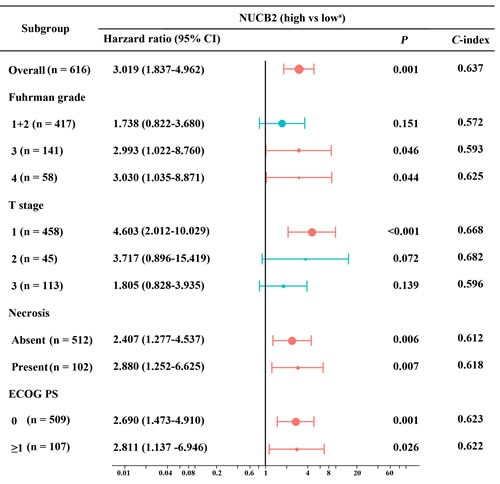
Subgroup analysis of NUCB2 expression for CSS among patients with different clinicopathological stratification Results expressed using hazard ratios. P value was two tailed. *C*-index was Harrell's concordance index. ^a^ Reference group.

In order to further assess the prognostic value of NUCB2 expression in different subgroup of patients, we combined the training cohort and validation cohort together, and calculated the hazard ratio and *C*-index of NUCB2 in different clinicopathological factor including pT stage, Fuhrman grade, ECOG PS and tumor necrosis. As indicated in Figure 3, NUCB2 expression was significantly associated with CSS of the patients with pT1 stage (HR 4.603, 95% CI 2.012-10.029, *P <* 0.001), while NUCB2 expression was of no statistical significance in patients with pT2 and pT3 stage. Interestingly, the NUCB2 expression in patients with Fuhrman grade 1+2 was not statistically associated with CSS while it was significantly correlated with CSS of the patients with Fuhrman grade 3 and 4 (HR 2.993, 95% CI 1.022-8.760, *P =* 0.046; HR 3.030, 95% CI 1.035-8.871, *P =* 0.044, respectively).

### Risk stratification for pT1N0M0 patients based on NNF model

**Figure 4:**
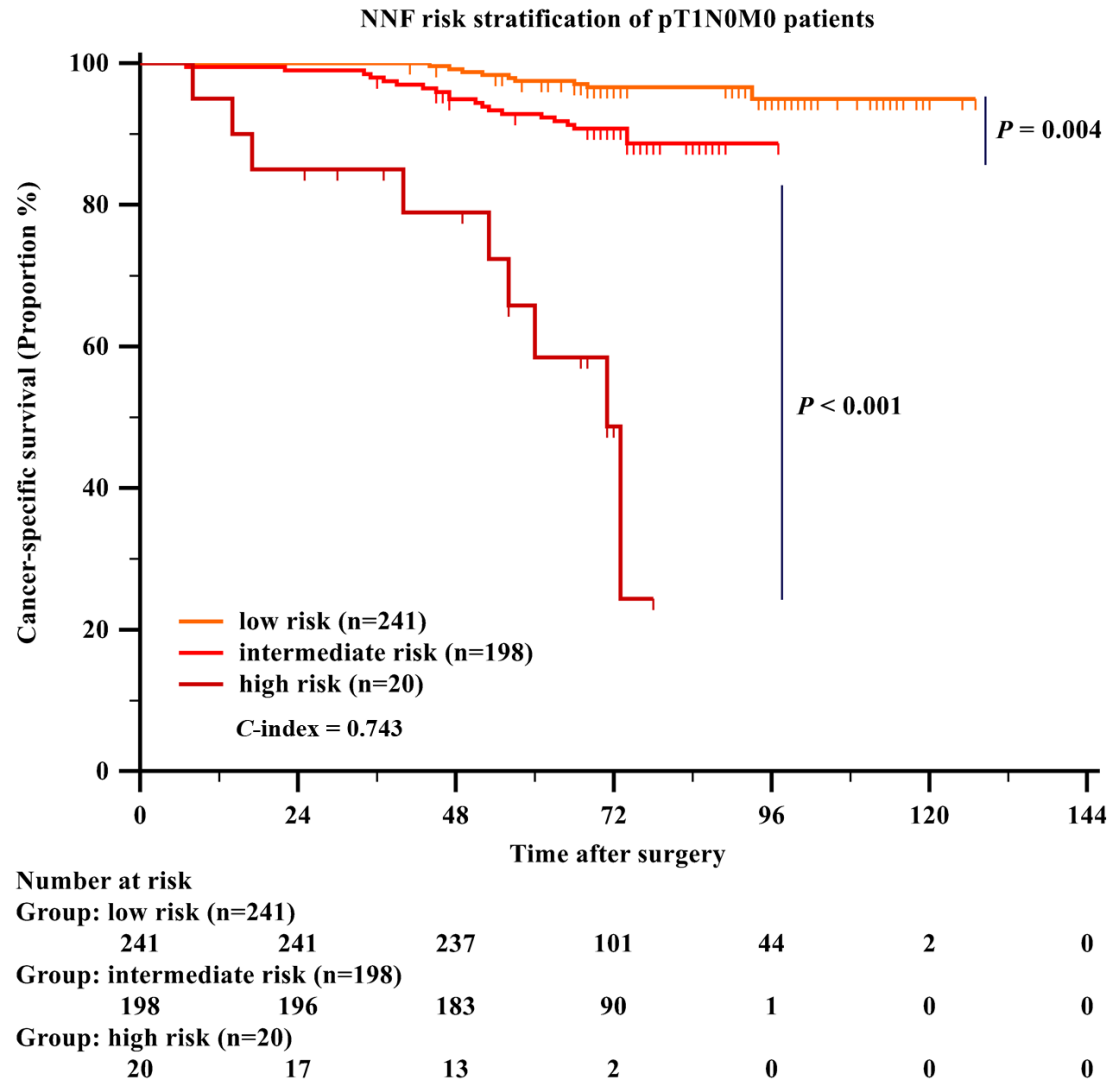
Kaplan-Meier analysis of cancer-specific survival of pT1N0M0 patients with clear-cell renal cell carcinoma (ccRCC) based on NNF risk stratification models P value was two tailed. *C*-index was Harrell's concordance index.

Based on the result of Figure 3, multivariate Cox regression was performed specifically in the pT1N0M0 patients. Tumor necrosis, Fuhrman grade and NUCB2 expression level was identified as independent prognostic factors. A new model was made: [NNF score = Necrosis score + NUCB2 score + Fuhrman score. Necrosis score (0: necrosis absent;4.5: necrosis present), NUCB2 score (0: low expression; 5.5: high expression) and Fuhrman score (0: Fuhrman grade 1+2; 3: Fuhrman grade3; 5: Fuhrman grade 4), Low risk: Total score ≤ 5, Intermediate risk: 5 < Total score ≤10.5, High risk: Total score > 10.5]. As shown in Figure 4, CSS could be significantly stratified through the new NNF stratification model (*P <* 0.001, C-index = 0.743).

## DISCUSSION

In this study, we illustrated that an increased NUCB2 expression was independently correlated with poor CSS for non-metastatic ccRCC patients in two different cohorts. The mRNA transcription level data from TCGA database also validated our conclusion. In addition, with the combination of NUCB2 expression as a dichotomized variable and established prognosis models like pT stage, SSIGN, UISS and Leibovich score algorithm, the predictive accuracy for CSS was observed obviously. Also, the dichotomized NUCB2 expression level stratified patients statistically significantly in pT1 subgroup of patients. Therefore, new NNF risk stratification model was developed to predict the prognosis of pT1N0M0 patients.

To the best of our knowledge, only one study addresses the prognostic significance of NUCB2 expression level in ccRCC using a multivariable Cox proportional analysis. In this study, Qi et al analyzed a cohort with 188 ccRCC patients and reported that high NUCB2 expression was statistically significantly correlated to pT stage and metastasis. However, in the immunoblot cohort of our study, such findings were not observed, which might due to the distinctive clinicopathological profile of studied patients. Multivariate Cox proportional analysis identified that high expression level of NUCB2 remain an independent unfavorable prognostic factor for OS in their relatively small cohort [20]. In this large validation study that included 616 patients with non-metastasis ccRCC from two distinctive medical institutions, we were able to clarify that a high NUCB2 expression level was an independent negative prognostic indicator for CSS. The NUCB2 mRNA transcription level was shown to be related to unfavorable prognosis for OS in TCGA KIRC cohort as well. Our findings corroborate the results of Qi et al who also found that NUCB2 expression level was an independent prognostic predictor in 188 RCC paitents with regard to OS.

Additionally, NUCB2 expression level was found to be statistically significantly associated with higher Fuhrman grade. Subgroup analysis also indicated that the prognostic value of NUCB2 expression was only significant in patients with Fuhrman grade 3 and 4. This result indicated that NUCB2 might play pivotal role in worse differentiated RCC development. Subgroup analysis also revealed that NUCB2 expression level could significantly identified the clinical outcome of pT1 stage patients. Therefore, we built a new NNF model to stratify different risk of pT1N0M0 patients which showed promising predictive accuracy. In the current days, the patients with pT1N0M0 stage consist the largest subgroup of RCC patients with the benefit of CT screening popularization. Multiple methods such as partial nephrectomy, radical nephrectomy and ablation were performed to these patients without certain consistency. Normally, tumor size was applied to differentiate T1a and T1b stage. Nephron-spare nephrectomy was recommended in T1a patients. However, what kinds of surgery should be performed in T1b patients is still controversial. The NNF score algorithm, which does not base on the tumor size, might guide the treatment option in this subgroup patients. Also, frequently surveillance might also be required in the higher risk NNF model subgroup patients of both T1a and T1b stage.

NUCB2/Nesfatin-1 showed widespread expression in the body, where it was mainly reported to participate in multiple pathophysiological processes like nocturnal feeding and body weight regulation [10]. Recently, a few studies revealed the correlation between NUCB2 and tumor development and possible mechanism. Takagi et al reported the NUCB2 expression was positively associated with Ki67 expression, and knockdown of NUCB2 significantly impaired tumor cell proliferation and migration in endometrial carcinoma [22]. Similarly, Kan et al found that nesfatin-1/NUCB-2 enhanced migration, invasion and EMT in colon cancer cells which might be involved in LKB1/AMPK/TORC1/ZEB1 pathway [17]. In this study, we analyzed the proteins significantly correlated to NUCB2 expression from TCGA KIRC cohort and identified the top 20 proteins in Table S1. These proteins are then integrated in STRING v10 protein-protein interaction networks to further investigate the possibly involved functional pathway [23]. It was shown to be participated in immune response-regulating cell surface receptor signaling pathway, cell cycle regulation and cell growth receptor signaling pathway with proteins involved like Caspase-8, PTEN, ERBB-2 and BCL-2 (Figure S2).

The limitation of our study included the retrospective data collection as with all retrospective studies. Also, surgical treatments of the patients were performed by multiple surgeons. Nonetheless, even considering these limitations, we clearly identified that high NUCB2 expression level is an independent prognostic indicator for CSS in non-metastatic ccRCC patients. Future prognostic studies should take this molecular marker into consideration and might integrate it into the established prognostic models.

In conclusion, we demonstrated that high expression level of NUCB2 was significantly associated with poor clinical outcome of patient with ccRCC. Moreover, NUCB2 could stratified patients in pT1 stage significantly and a new NNF model could effectively stratified different risk patients in pT1N0M0 stage. This molecular marker should be considered in adjuvant trials and future risk assessment tools as a selection criterion for risk factors-stratified patient management in non-metastatic ccRCC.

## SUPPLEMENTARY MATERIALS FIGURES AND TABLES


