# Supplementary material for: High NUCB2 expression level represents an independent negative prognostic factor in Chinese cohorts of non-metastatic clear cell renal cell carcinoma patients
Source: Oncotarget. 2016 Oct 28;8(21):35244–54. doi: 10.18632/oncotarget.12961 (PMC5471050; doi:10.18632/oncotarget.12961)
Supplement: Supplementary file 1 [file oncotarget-08-35244-s001.pdf]

High NUCB2 expression level represents an independent negative prognostic factor in Chinese cohorts of non-metastatic clear cell renal cell carcinoma patients

Supplementray Material

IHC protocol:

Kidney TMA and tissue sections were incubated overnight at 60 °C followed by deparaffinization using repeated washes of xylene (15 min each, 60 °C) followed by rehydration of tissues with graded alcohol. After rehydration, tissues were incubated with methanolic solution of 3% H2O2 for quenching of endogenous peroxidase activity. Heat induced antigen retrieval was performed heating the sample in 0.01M citrate (pH 8.0, 100°C, pH=6) buffer for 15 min in microwave oven. Following antigen retrieval, the sections were blocked with goat serum (Origene) for 30 minutes at room temperature. The slides were further incubated with rabbit anti-NUCB2 polyclonal antibody (Sigma, 1:100) overnight at 4°C. After four washes with TBS-T (TBS containing 0.05% Tween 20), the sections were incubated with anti-mouse secondary antibody ((Origene substrate kit; Origene)) for 30 min. and subsequently, the color was developed by adding 3, 39-diaminobenzidine solution (Origene substrate kit; Origene). Reddish brown precipitate indicated positive immuno-reactivity. The slides were counterstained with haematoxylin, dehydrated in graded ethanol followed by xylene and mounted. All slides were analyzed using Nikon Eclipse Ti Microscope (Nikon Corporation, Tokyo, Japan) and Leica DM6000 B (Leica Microsystems, Wetzlar, Germany).

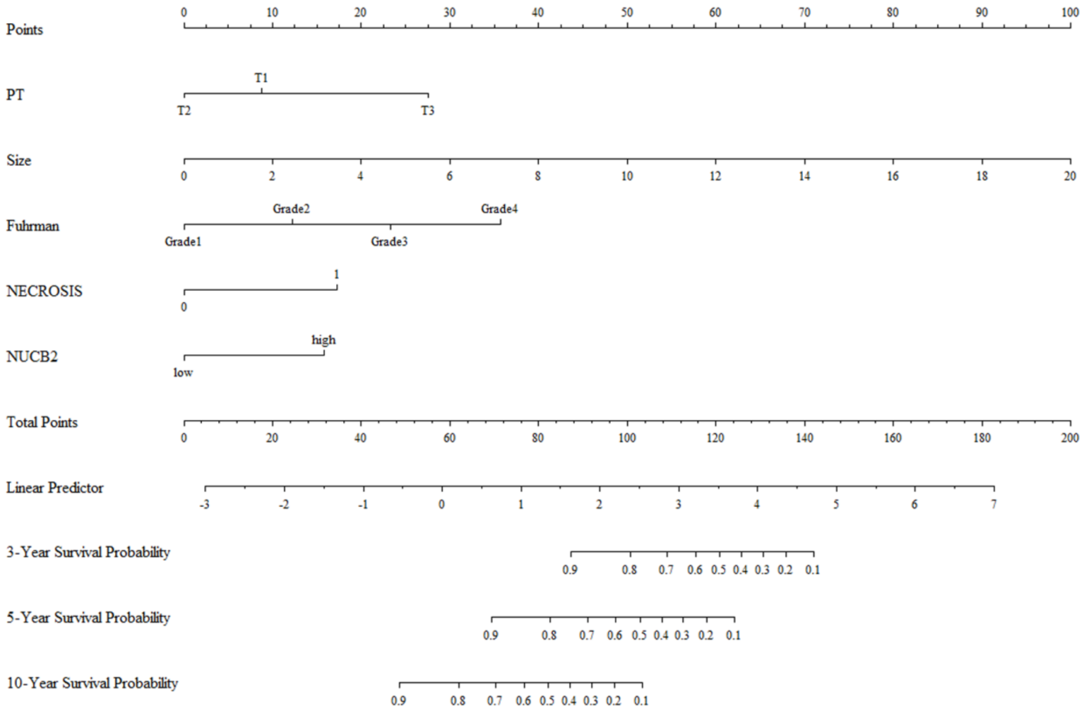

Figure S1. Nomogram for prediction of clinical outcomes in patients with clear-cell renal cell carcinoma (ccRCC).

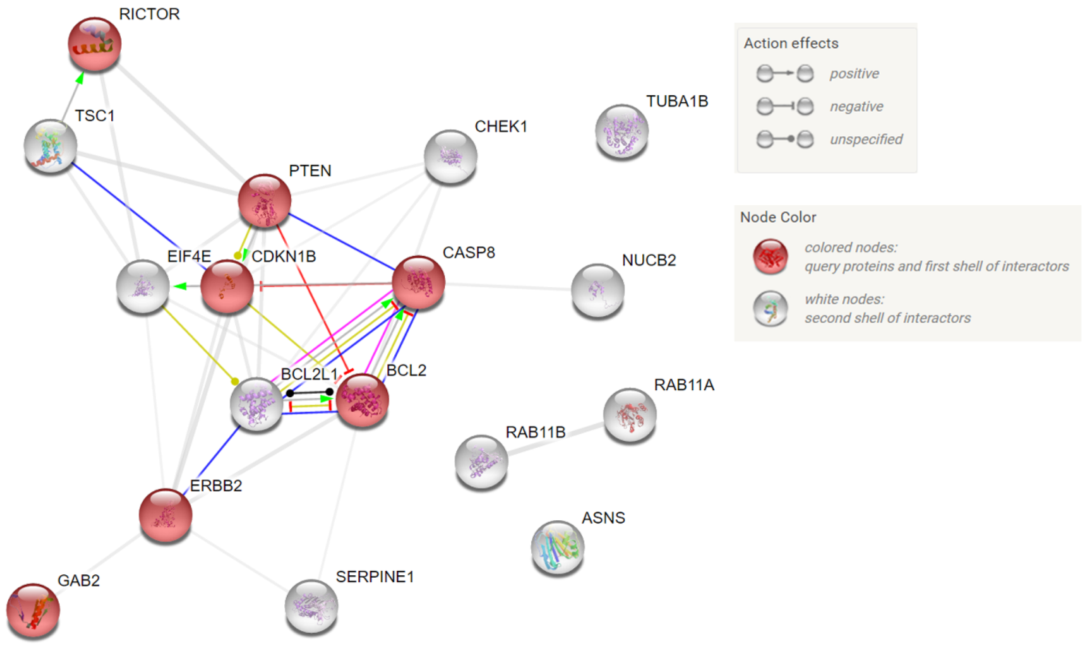

Figure S2. STRING v10 protein-protein interaction networks.

Table S1. Correlation between phosphoprotein / Protein expression (RPPA) of NUCB2 and other proteins from TCGA KIRC cohort.

Gene Name	Cytoband	Mean protein expression(Altered)	Mean protein expression(Unaltered)	SD of protein expression(Altered)	SD of protein expression(Unaltered)	*P*	q-Value
RICTOR	5p13.1	-0.44	0.12	0.6	0.87	8.12E-05	9.19E-03
RAB11B	19p13.2	0.3	0.64	0.37	0.53	1.13E-04	9.19E-03
RAB11A	15q22.31	0.3	0.64	0.37	0.53	1.13E-04	9.19E-03
PRKAA1_PT172	5p12	-0.6	-0.09	0.61	0.62	3.13E-04	0.0191
ASNS	7q21.3	-0.04	-0.7	0.86	0.51	5.81E-04	0.0278
GAB2	11q14.1	0.09	0.64	0.71	0.88	6.83E-04	0.0278
CASP8	2q33-q34	0.42	0.26	0.22	0.18	1.48E-03	0.0486
CDKN1B	12p13.1-p12	-0.04	0.15	0.27	0.27	1.74E-03	0.0486
TSC1	9q34	-0.29	0.11	0.58	0.34	1.84E-03	0.0486
PTEN	10q23.3	-0.29	0.15	0.66	0.4	2.09E-03	0.0486
STAT3_PY705	17q21.31	-0.23	0	0.34	0.44	2.19E-03	0.0486
TUBA1B	12q13.12	-0.79	-0.15	0.99	0.72	2.93E-03	0.0556
ERBB2	17q12	-0.52	-0.24	0.42	0.31	2.96E-03	0.0556
EIF4E	4q23	-0.18	-0.32	0.22	0.21	4.85E-03	0.0811
CHEK1	11q24.2	0.04	-0.11	0.24	0.16	4.99E-03	0.0811
BCL2L1	20q11.21	0.04	-0.18	0.36	0.28	5.88E-03	0.0896
BCL2	18q21.3	0.17	0.46	0.5	0.39	6.33E-03	0.0909
SERPINE1	7q22.1	1.64	0.74	1.55	1.02	7.52E-03	0.102

SD, Standard deviation.

[a]$P<0.05$ is considered statistically significant.
